# COVID-19 Severity Is Associated with Differential Antibody Fc-Mediated Innate Immune Functions

**DOI:** 10.1128/mBio.00281-21

**Published:** 2021-04-20

**Authors:** Opeyemi S. Adeniji, Leila B. Giron, Mansi Purwar, Netanel F. Zilberstein, Abhijeet J. Kulkarni, Maliha W. Shaikh, Robert A. Balk, James N. Moy, Christopher B. Forsyth, Qin Liu, Harsh Dweep, Andrew Kossenkov, David B. Weiner, Ali Keshavarzian, Alan Landay, Mohamed Abdel-Mohsen

**Affiliations:** a The Wistar Institute, Philadelphia, Pennsylvania, USA; b Rush University, Chicago, Illinois, USA; Indiana University Bloomington

**Keywords:** SARS-CoV-2, COVID-19, antibody, Fc-mediated functions, inflammation

## Abstract

Beyond neutralization, antibodies binding to their Fc receptors elicit several innate immune functions including antibody-dependent complement deposition (ADCD), antibody-dependent cell-mediated phagocytosis (ADCP), and antibody-dependent cell-mediated cytotoxicity (ADCC). These functions are beneficial, as they contribute to pathogen clearance; however, they also can induce inflammation. We tested the possibility that qualitative differences in SARS-CoV-2-specific antibody-mediated innate immune functions contribute to coronavirus disease 2019 (COVID-19) severity. We found that anti-S1 and anti-RBD antibodies from hospitalized COVID-19 patients elicited higher ADCD but lower ADCP compared to antibodies from nonhospitalized COVID-19 patients. Consistently, higher ADCD was associated with higher systemic inflammation, whereas higher ADCP was associated with lower systemic inflammation during COVID-19. Our study points to qualitative, differential features of anti-SARS-CoV-2 specific antibodies as potential contributors to COVID-19 severity. Understanding these qualitative features of natural and vaccine-induced antibodies will be important in achieving optimal efficacy and safety of SARS-CoV-2 vaccines and/or COVID-19 therapeutics.

## Abstract (precis)

A state of hyperinflammation and increased complement activation has been associated with coronavirus disease 2019 (COVID-19) severity. However, the pathophysiological mechanisms that contribute to this phenomenon remain mostly unknown.

## OBSERVATION

While most SARS-CoV-2-infected individuals exhibit asymptomatic or mild respiratory tract infection, a significant population face severe symptoms requiring hospitalization (1, 2). Hospitalized coronavirus disease 2019 (COVID-19) is associated with a state of hyperinflammation and increased complement activation (1, 3–5). However, the mechanisms that contribute to this hyperinflammation are not fully clear.

Higher titers of SARS-CoV-2-specific neutralizing antibodies have been associated with higher COVID-19 severity (6, 7). However, beyond neutralization, antibodies can elicit an array of Fc-mediated innate immune functions such as antibody-dependent complement deposition (ADCD), antibody-dependent cellular phagocytosis (ADCP), and antibody-dependent cell-mediated cytotoxicity (ADCC) (8). These innate immune functions are beneficial, as they contribute to pathogen clearance; however, they also can induce inflammation during viral infections (8). Here, we hypothesized that differential, qualitative features of SARS-CoV-2-specific antibodies contribute to COVID-19 severity. To test this hypothesis, we examined the ability of S1- and RBD-specific immunoglobulin G (IgG) from hospitalized and nonhospitalized COVID-19 patients (and controls) (Table S1) to elicit ADCD, ADCP, and ADCC.

## 

### Severe COVID-19 is associated with higher ADCD and lower ADCP compared to mild COVID-19.

**FIG 1:**
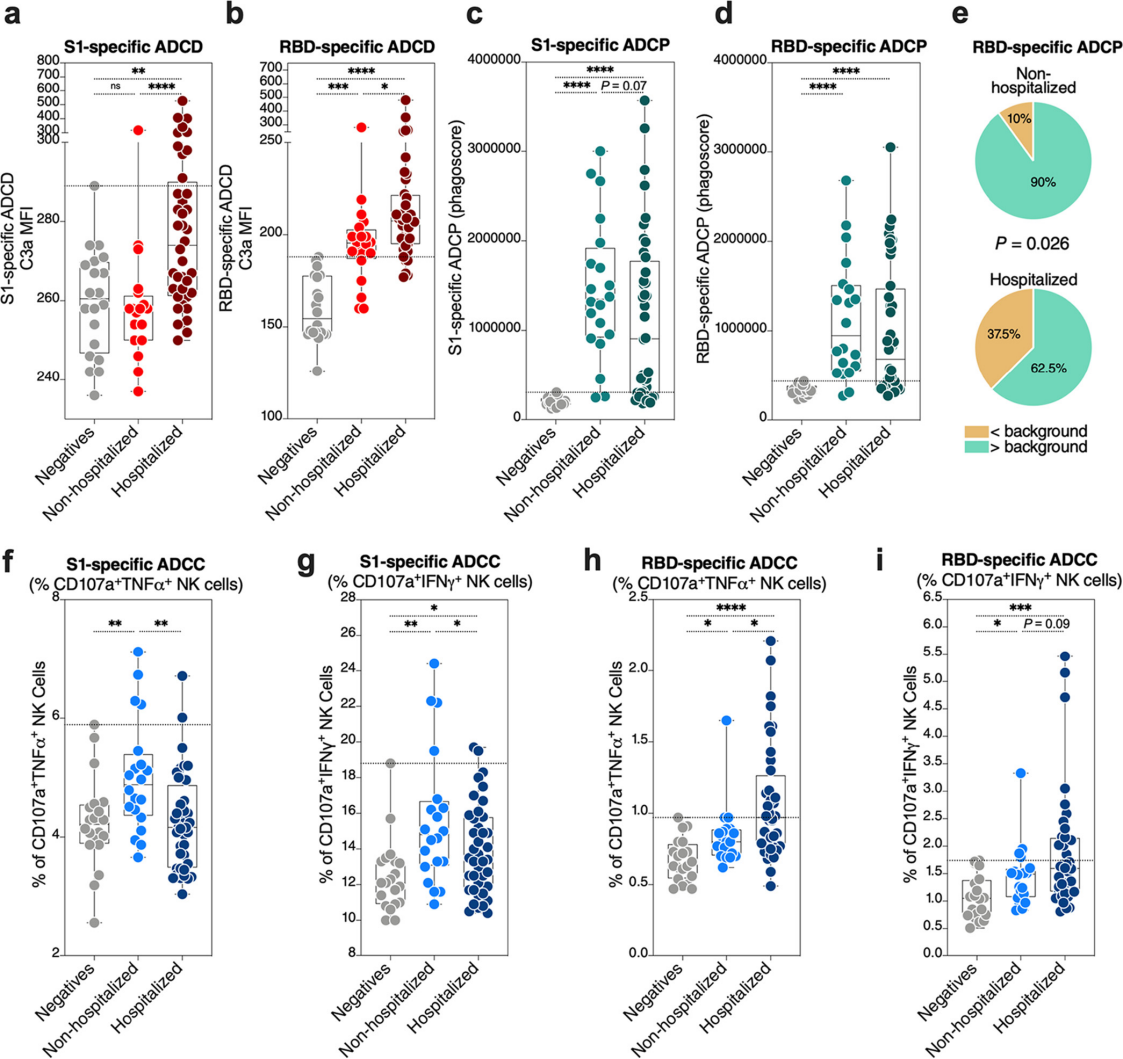
Hospitalized COVID-19 is associated with differential antibody Fc-mediated innate immune functions. (a and b) The ability of anti-S1 (a) and anti-RBD (b) antibodies to elicit antibody-dependent complement deposition (ADCD) was measured as the ability of 1 mg/ml of IgG to fix complement on target cells. The Kruskal-Wallis test was used for statistical analysis. Horizontal dotted lines indicate the maximum values from the SARS-CoV-2 negative-control group. (c and d) The ability of anti-S1 (c) and anti-RBD (d) antibodies to elicit antibody-dependent cellular phagocytosis (ADCP) was measured as the ability of 1 mg/ml of IgG to mediate phagocytosis of S1 and RBD antigen-coated beads. The Kruskal-Wallis test was used for statistical analysis. Horizontal dotted lines indicate the maximum values from the SARS-CoV-2 negative-control group. (e) Comparison between the fraction of nonhospitalized and hospitalized COVID-19 patients whose antibodies elicit anti-RBD-specific ADCP with values higher than background (maximum value from the SARS-CoV-2 negative-control group). The chi-square test was used for statistical analysis. (f to i) The ability of anti-S1 (f and g) and anti-RBD (h and i) antibodies to elicit antibody-dependent cell-mediated cytotoxicity (ADCC) was estimated by NK cell degranulation and intracellular cytokine (TNF-α [f and h] or IFN-γ [g and i]) production after exposure of 100 μg/ml of IgG to S1- and RBD-pulsed target cells. The Kruskal-Wallis test was used for statistical analysis. Horizontal dotted lines indicate the maximum values from the SARS-CoV-2 negative-control group. *, *P* < 0.05; **, *P* < 0.01; ***, *P* < 0.001; ****, *P* < 0.0001; ns, not significant.

IgG from hospitalized COVID-19 patients elicited significantly higher ADCD against S1- and RBD-coated target cells compared to IgG from nonhospitalized individuals (Fig. 1a and b). In contrast, a higher fraction of IgG from nonhospitalized COVID-19 patients elicited ADCP activity above background (maximum value from the SARS-CoV-2-negative group) compared to hospitalized patients (Fig. 1c to e). S1-specific antibodies from hospitalized individuals induced significantly higher NK cell degranulation and intracellular cytokine production (a surrogate of ADCC) than did S1-specific antibodies from nonhospitalized individuals (Fig. 1f and g). In contrast, RBD-specific antibodies from nonhospitalized individuals induced significantly higher ADCC surrogates than did RBD antibodies from hospitalized individuals (Fig. 1h and i). These data suggest that hospitalized COVID-19 is associated with differential, qualitative features of SARS-CoV-2-specific antibodies. In particular, hospitalized COVID-19 is associated with a higher ability of antibodies to elicit complement deposition and a lower ability to elicit phagocytosis. These data are compatible with reports suggesting that complement immune system plays a significant negative role in coronavirus disease pathogenesis (9–11).

### SARS-CoV-2 antibody titers correspond to ADCP but not ADCD or ADCC.

**FIG 2:**
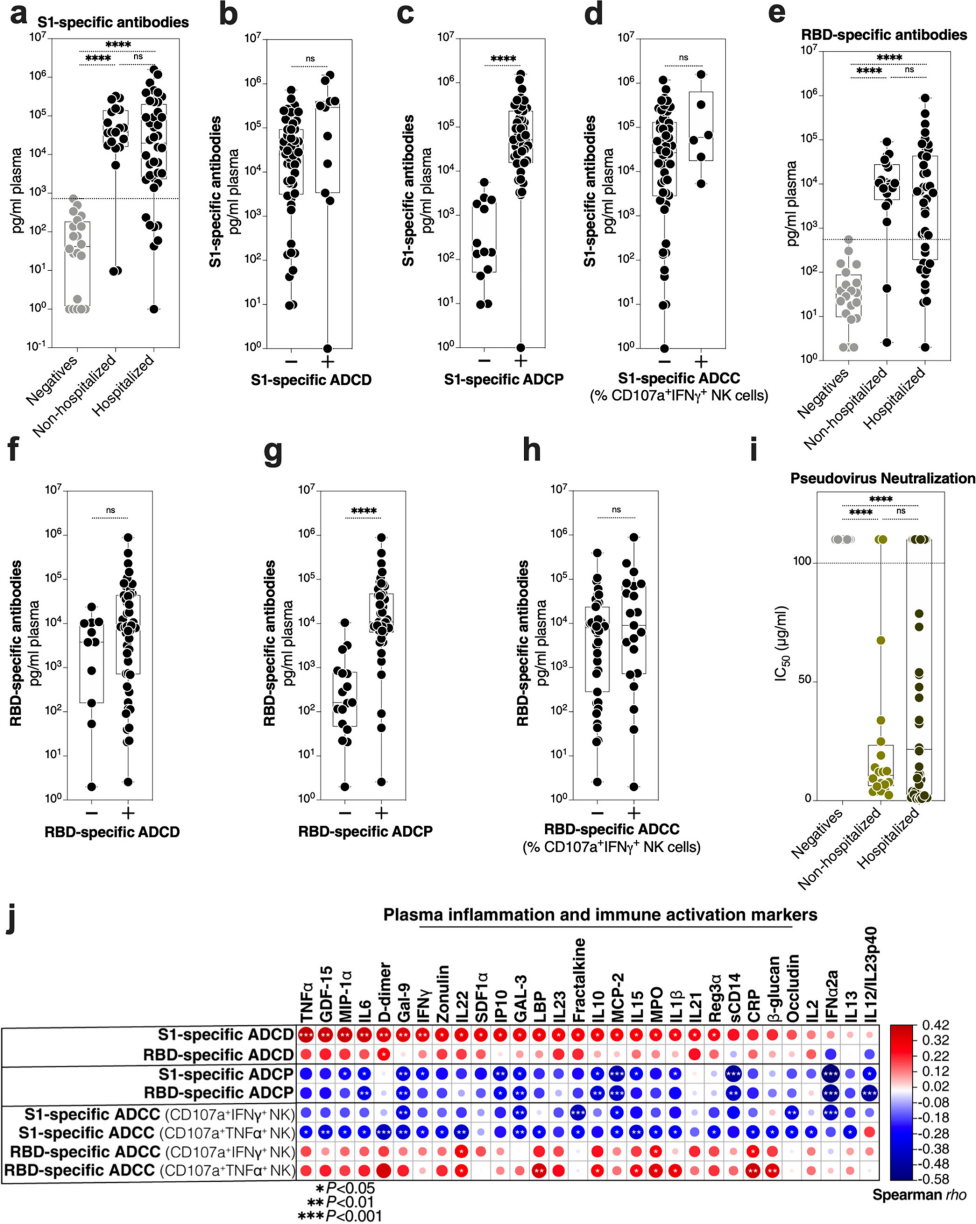
Antibody titers do not correlate with ability to elicit ADCD, which associates with higher systemic inflammation. (a) S1-specific antibody titers of the indicated groups. The Kruskal-Wallis test was used for statistical analysis. The horizontal dotted line indicates the maximum value from the SARS-CoV-2 negative-control group. (b to d) S1-specific antibody titers in individuals whose antibodies can elicit S1-specific ADCD (b), ADCP (c), or ADCC (d) higher (+ symbol) or lower (− symbol) than background (maximum value from the SARS-CoV-2 negative-control group). The Mann-Whitney U test was used in statistical analysis. (e) RBD-specific antibody titers of the indicated groups. The Kruskal-Wallis test was used for statistical analysis. The horizontal dotted line indicates the maximum value from the SARS-CoV-2 negative-control group. (f to h) RBD-specific antibody titers in individuals whose antibodies can elicit RBD-specific ADCD (f), ADCP (g), or ADCC (h) higher (+ symbol) or lower (− symbol) than background (maximum value from the SARS-CoV-2 negative-control group). The Mann-Whitney U test was used in statistical analysis. (i) The ability of purified IgG to neutralize SARS-CoV-2 pseudovirus. Total IgG concentrations were normalized across samples and ranged from 100 μg/ml to 0.045 μg/ml to yield an IC_50_. Any sample that yielded no measured neutralization ability was given a value of 110 μg/ml for graphing purposes. The Kruskal-Wallis test was used for statistical analysis. (j) Spearman's correlation heat map showing associations between Fc-mediated innate immune functions in rows and levels of plasma inflammatory markers in columns during COVID-19 (*n* = 60). The size and color of circles represent the strength of the correlation, with blue shades representing negative correlations and red shades representing positive correlations. *, *P* < 0.05; **, *P* < 0.01; ***, *P* < 0.001; ****, *P* < 0.0001; ns, not significant.

We next sought to examine whether the differential levels of ADCD, ADCP, and ADCC between hospitalized and nonhospitalized patients can be simply explained by the titers of anti-S1 and anti-RBD antibodies. There was no significant difference in the titer of S1-specific antibodies between hospitalized and nonhospitalized patients in our cohort (Fig. 2a). When we examined S1-specific antibody titers in patients having ADCD, ADCP, or ADCC values below or above background levels (defined as the maximum values observed in the SARS-CoV-2-negative group), we found no difference in ADCD or ADCC but a significant difference in ADCP (Fig. 2b to d). Similar results were observed with RBD-specific antibodies (Fig. 2e to h). We also examined the ability of these IgGs to neutralize SARS-CoV-2 pseudovirus. There was no significant difference between the neutralization abilities of IgG from nonhospitalized and hospitalized patients in our cohort (Fig. 2i). However, half maximal inhibitory concentrations (IC_50_s) from the neutralization assay strongly correlated with higher ADCP (the higher the ability of IgGs to neutralize SARS-CoV-2 pseudovirus, the higher their ability to elicit ADCP) (see Fig. S1 in the supplemental material). These data indicate that the quantities of S1- or RBD-specific antibodies could explain the differences in ADCP activity between hospitalized and nonhospitalized patients (the higher the antibody titer, the higher the ADCP activity) but cannot fully explain the differences in ADCD or ADCC activity between hospitalized and nonhospitalized patients, suggesting that qualitative rather than quantitative properties of anti-SARS-CoV-2 antibodies associate with disease severity.

10.1128/mBio.00281-21.1 FIG S1 Correlations between the ability of IgGs to neutralize SARS-CoV-2 pseudovirus and antibody-mediated innate immune functions. Spearman’s rank correlation tests were used for statistical analyses. Download FIG S1, PDF file, 0.4 MB. Copyright © 2021 Adeniji et al. 2021 Adeniji et al. https://creativecommons.org/licenses/by/4.0/ This content is distributed under the terms of the Creative Commons Attribution 4.0 International license.

### Anti-SARS-CoV-2 ADCD associates with higher inflammation.

Given that complement activation can significantly contribute to inflammation-mediated tissue damage, including during COVID-19 (12, 13), we examined the relationship between the ADCD property of IgGs (as well as ADCP and ADCC) and 39 plasma markers of systemic inflammation and immune activation (a list is in Table S2). As expected, higher S1- and RBD-specific ADCD was associated with higher levels of several markers of inflammation and immune activation (Fig. 2j). In contrast, higher levels of S1- and RBD-specific ADCP were associated with lower levels of several inflammatory markers (Fig. 2j). Finally, ADCC exhibited an antigen-specific relationship with inflammation: anti-S1 specific ADCC exhibited negative correlations with inflammation, whereas anti-RBD exhibited positive correlations with inflammation (Fig. 2j). These data further point to the potentially detrimental effects of ADCD on COVID-19 pathogenesis.

10.1128/mBio.00281-21.6 TABLE S2 A list of plasma markers measured in this study. Download Table S2, PDF file, 0.04 MB. Copyright © 2021 Adeniji et al. 2021 Adeniji et al. https://creativecommons.org/licenses/by/4.0/ This content is distributed under the terms of the Creative Commons Attribution 4.0 International license.

Future studies will be needed to examine other antigen-specific antibodies including anti-N antibodies, as well as different antibody classes such as IgA and IgM. These studies will also need to examine the mechanisms that underlie these differential, qualitative features. Antibody glycosylation and subclass have been shown as determinants of Fc-mediated innate immune functions (14). COVID-19 has been associated with high levels of proinflammatory IgG glycomic structures (15). Understanding how these glycomic features impact the different antibody-mediated innate immune functions during COVID-19 will be needed. Our sample size did not allow for addressing potential confounders. However, we observed no difference in antibody-mediated innate immune functions in individuals who received hydroxychloroquine, tocilizumab, or steroids compared with individuals who did not (Fig. S2, S3, and S4, respectively). Independent test sets from large cohorts from various geographic and demographic settings (including a wider range in age) will be needed in future studies. Finally, it will be important to understand how these antibody features impact the function of vaccine-mediated antibodies as well as therapeutic antibodies in order to achieve an optimal balance between neutralization and innate immune functions without inciting potential side effects.

10.1128/mBio.00281-21.2 FIG S2 Differences in antibody-mediated innate immune functions in hospitalized patients who received hydroxychloroquine versus patients who did not. The Mann-Whitney U test was used in statistical analysis. Download FIG S2, PDF file, 0.4 MB. Copyright © 2021 Adeniji et al. 2021 Adeniji et al. https://creativecommons.org/licenses/by/4.0/ This content is distributed under the terms of the Creative Commons Attribution 4.0 International license.

10.1128/mBio.00281-21.3 FIG S3 Differences in antibody-mediated innate immune functions in hospitalized patients who received tocilizumab versus patients who did not. The Mann-Whitney U test was used in statistical analysis. Download FIG S3, PDF file, 0.4 MB. Copyright © 2021 Adeniji et al. 2021 Adeniji et al. https://creativecommons.org/licenses/by/4.0/ This content is distributed under the terms of the Creative Commons Attribution 4.0 International license.

10.1128/mBio.00281-21.4 FIG S4 Differences in antibody-mediated innate immune functions in hospitalized patients who received steroids versus patients who did not. The Mann-Whitney U test was used in statistical analysis. Download FIG S4, PDF file, 0.4 MB. Copyright © 2021 Adeniji et al. 2021 Adeniji et al. https://creativecommons.org/licenses/by/4.0/ This content is distributed under the terms of the Creative Commons Attribution 4.0 International license.

In summary, our data suggest that differential, qualitative Fc-mediated antibody effector properties of anti-SARS-CoV-2 antibodies associate with COVID-19 severity. In particular, antibodies in individuals hospitalized with COVID-19 elicit higher levels of complement deposition compared to antibodies in individuals with mild COVID-19, in a manner linked to higher systemic inflammation. Understanding these qualitative properties will be important to develop COVID-19 therapeutics and SARS-CoV-2 vaccines with optimal efficacy and safety.

### Ethics.

All research protocols of the study were approved by the institutional review boards (IRB) at Rush University and The Wistar Institute. All human experimentation was conducted in accordance with the guidelines of the U.S. Department of Health and Human Services and those of the authors’ institutions.

### Characteristics of the study cohort.

We isolated IgG antibodies (using Pierce Protein G Spin Plate; Thermo Fisher) from 60 individuals who tested positive for SARS-CoV-2 (by PCR) and 20 negative controls. The 60 SARS-CoV-2-positive individuals were either outpatients (nonhospitalized; *n* = 20) or inpatients (hospitalized; *n* = 40) (Table S1). Individuals were selected to have a median age between 52.5 and 58.5 years to avoid age bias in disease outcome. The cohort was also chosen to be 45 to 60% female per group. Samples from hospitalized patients were collected when the patient was admitted. Samples from nonhospitalized patients were collected a median of 27 days after onset of COVID-19 symptoms. The concentration of purified IgG antibodies was determined using NanoDrop (absorbance at *A*_280_). Purified IgG antibodies were diluted with phosphate-buffered saline (PBS) and adjusted to a 1-mg/ml concentration which was used in ADCD, ADCP, ADCC, and neutralization assays.

10.1128/mBio.00281-21.5 TABLE S1 Demographic and clinical characteristics of the study cohort. Download Table S1, PDF file, 0.05 MB. Copyright © 2021 Adeniji et al. 2021 Adeniji et al. https://creativecommons.org/licenses/by/4.0/ This content is distributed under the terms of the Creative Commons Attribution 4.0 International license.

### ADCD measurements.

Purified IgG antibodies were analyzed for their ability to fix complement on target cells. (ACE2)-CHO cells (4 × 10^6^) were pulsed with 20 μg biotinylated SARS-CoV-2 S1 or RBD proteins (Acro Biosystems) for 30 min at 37°C. Excess, unbound antigens were removed by washing cells once with complete medium. Twenty micrograms of IgG was added to the antigen-pulsed cells and incubated for another 30 min at 37°C. Freshly resuspended lyophilized guinea pig complement (Cedarlane) diluted 1:20 with Veronal buffer, 0.1% gelatin with calcium and magnesium (Boston BioProducts), was added to the cells for 2 h at 37°C. Following a wash with 1× PBS, cells were assessed for complement deposition by staining with goat anti-guinea pig C3a-fluorescein isothiocyanate (FITC) (MP Biomedicals). After fixing, cells were analyzed by flow cytometry, and ADCD is reported as median fluorescence intensity (MFI) of FITC^+^ cells.

### ADCP measurements.

Purified plasma IgG antibodies were analyzed for their ability to mediate phagocytosis of S1 and RBD antigen-coated beads. Biotinylated SARS-CoV-2 S1 and RBD proteins were combined with fluorescent Neutravidin beads (Life Technologies) overnight at 4°C. Excess, unconjugated antigens were removed by washing the beads twice with 0.1% PBS-bovine serum albumin (BSA). Beads were washed with 1 ml buffer and spun at 14,000 × *g* for 2 min at room temperature. Antigen-coated beads were resuspended in a final volume of 1 ml in PBS-BSA. Beads (10 μl) were added into each well of a round-bottom 96-well culture plate, after which 20 μg IgG was added to each well and incubated for 2 h at 37°C. A 200 μl suspension of THP-1 cells at 2.5 × 10^5^ cells/ml was added to each well, for a total of 5 × 10^4^ THP-1 cells per well. After mixing, the cell-bead mixtures were incubated overnight at 37°C. The following day, 100 μl of supernatant from each well was removed, and 100 μl of BD Cytofix was added to each well. Cells were analyzed by flow cytometry, and data collected were analyzed in FlowJo software. Phagoscores were computed by multiplying the percentage of fluorescent bead^+^ cells by the median fluorescence intensity (MFI) of bead^+^ cells.

### ADCC measurements.

ADCC induction was estimated by NK cell degranulation and intracellular cytokine production after exposure of antibodies to S1- and RBD-pulsed target cells. S1- or RBD-pulsed (ACE2)-CHO cells (2.5 × 10^4^) were mixed with 20 μg IgG and incubated for 30 min at 37°C. Human NK cells (1 × 10^5^) isolated from peripheral blood mononuclear cells (PBMC) using the EasySep human NK cell isolation kit (Stem Cell Technologies) were added to wells in the presence of CD107a phycoerythrin (PE) (BD) and Golgi Stop (BD). Pulsed (ACE2)-CHO immune complexes were then added to the wells, mixed, pelleted, and incubated at 37°C for 16 h. Postincubation, NK cell activation was detected by staining for CD56 peridinin chlorophyll protein (PerCP)-Cy5.5 (BD), gamma interferon (IFN-γ) (BD), and tumor necrosis factor (TNF) (BioLegend) and acquired via flow cytometry. Data are reported as the percentage of cells positive for the marker as indicated.

### Measurement of S1- and RBD-specific antibody titers.

S1- and RBD-specific antibody titers were measured in the plasma of the 80 individuals using the MSD V‐Plex multiplex assay (Meso Scale Diagnostic).

### SARS-CoV-2 pseudovirus neutralization assay.

SARS-CoV-2-DeltaCT pseudovirus was produced using HEK-293T cells transfected with GeneJammer (Agilent) using IgE-SARS-CoV-2 S plasmid with a 19-amino-acid deletion at the C terminus (GenScript) and pNL4-3.Luc.R-E- plasmid (NIH AIDS reagent) at a 1:1 ratio. For setting up the pseudovirus neutralization assay, 10,000 (ACE2)-CHO cells per well were plated in 96-well plates in 100 μl medium and were rested at 37°C and 5% CO_2_ for 24 h. On the following day, purified IgGs were serially diluted (from 100 μg/ml to 0.045 μg/ml). IgGs were incubated with a fixed amount of SARS-CoV-2-DeltaCT pseudovirus for 90 min at room temperature. After incubation, the IgG-virus mixture was added to the plated cells and was incubated in a standard incubator (37% humidity, 5% CO_2_) for 72 h. After 72 h, cells were lysed using the Britelite Plus luminescence reporter gene assay system (Perkin-Elmer), and relative light units (RLU) were measured using a BioTek plate reader. The concentrations yielding 50% virus neutralization/inhibition (IC_50_) were calculated and defined as the sample concentration at which RLU are reduced by 50% compared to RLU in virus control wells after subtraction of background RLU in cell control wells.

### Measurement of plasma markers of inflammation.

Plasma levels of granulocyte-macrophage colony-stimulating factor (GM-CSF), IFN-β, IFN-γ, interleukin-10 (IL-10), IL-13, IL-1β, IL-33, IL-4, IL-6, TNF-α, fractalkine, IL-12p70, IL-2, IL-21, IL-22, IL-23, IP-10, MCP-2, MIP-1α, SDF-1a, IFN-α2a, IL-12/IL-23p40, and IL-15 were determined using a customized MSD U‐Plex multiplex assay (Meso Scale Diagnostic). Plasma levels of C-reactive protein (CRP), galectin-1, galectin-3, galectin-9, growth differentiation factor-15 (GDF-15), soluble CD14 (sCD14), soluble CD163 (sCD163), lipopolysaccharide (LPS) binding protein (LBP), and FABP2/I-FABP were measured using enzyme-linked immunosorbent assay (ELISA) kits (R&D Systems). The plasma level of zonulin was measured using an ELISA kit from MyBioSource. Levels of occludin were measured by ELISA (Biomatik). Plasma levels of Reg3A were measured by ELISA (RayBiotech). β-Glucan detection in plasma was performed using *Limulus* amebocyte lysate (LAL) assay (Glucatell kit; Associates of Cape Cod Inc.).

### Statistical analysis.

Kruskal-Wallis and Mann-Whitney U tests were used for unpaired comparisons. Chi-square test and Fisher's exact test were used for binary comparisons. Spearman’s rank correlations were used for bivariate correlation analyses. Statistical analyses were performed in Prism 7.0 (GraphPad).
