# Erratum for Adeniji et al., “COVID-19 Severity Is Associated with Differential Antibody Fc-Mediated Innate Immune Functions”

**DOI:** 10.1128/mBio.01244-21

**Published:** 2021-06-08

**Authors:** Opeyemi S. Adeniji, Leila B. Giron, Mansi Purwar, Netanel F. Zilberstein, Abhijeet J. Kulkarni, Maliha W. Shaikh, Robert A. Balk, James N. Moy, Christopher B. Forsyth, Qin Liu, Harsh Dweep, Andrew Kossenkov, David B. Weiner, Ali Keshavarzian, Alan Landay, Mohamed Abdel-Mohsen

**Affiliations:** a The Wistar Institute, Philadelphia, Pennsylvania, USA; b Rush University, Chicago, Illinois, USA

## ERRATUM

Volume 12, no. 2, e00281-21, 2021, https://doi.org/10.1128/mBio.00281-21. On PDF page 2, under “Severe COVID-19 is associated with higher ADCD and lower ADCP compared to mild COVID-19,” the following sentences are incorrect: “S1-specific antibodies from hospitalized individuals induced significantly higher NK cell degranulation and intracellular cytokine production (a surrogate of ADCC) than did S1-specific antibodies from nonhospitalized individuals (Fig. 1f and g). In contrast, RBD-specific antibodies from nonhospitalized individuals induced significantly higher ADCC surrogates than did RBD antibodies from hospitalized individuals (Fig. 1h and i).” Instead, these sentences should read as follows: “S1-specific antibodies from hospitalized individuals induced significantly lower NK cell degranulation and intracellular cytokine production (a surrogate of ADCC) than did S1-specific antibodies from nonhospitalized individuals (Fig. 1f and g). In contrast, RBD-specific antibodies from nonhospitalized individuals induced significantly lower ADCC surrogates than did RBD antibodies from hospitalized individuals (Fig. 1h and i).” This change does not change the conclusions of the paper.

