# Supplementary figures and images for: COVID-19 Severity Is Associated with Differential Antibody Fc-Mediated Innate Immune Functions
Source: mBio. 2021 Apr 20;12(2):e00281-21. doi: 10.1128/mBio.00281-21 (PMC8092230; doi:10.1128/mBio.00281-21)

Supplementary Figure 1

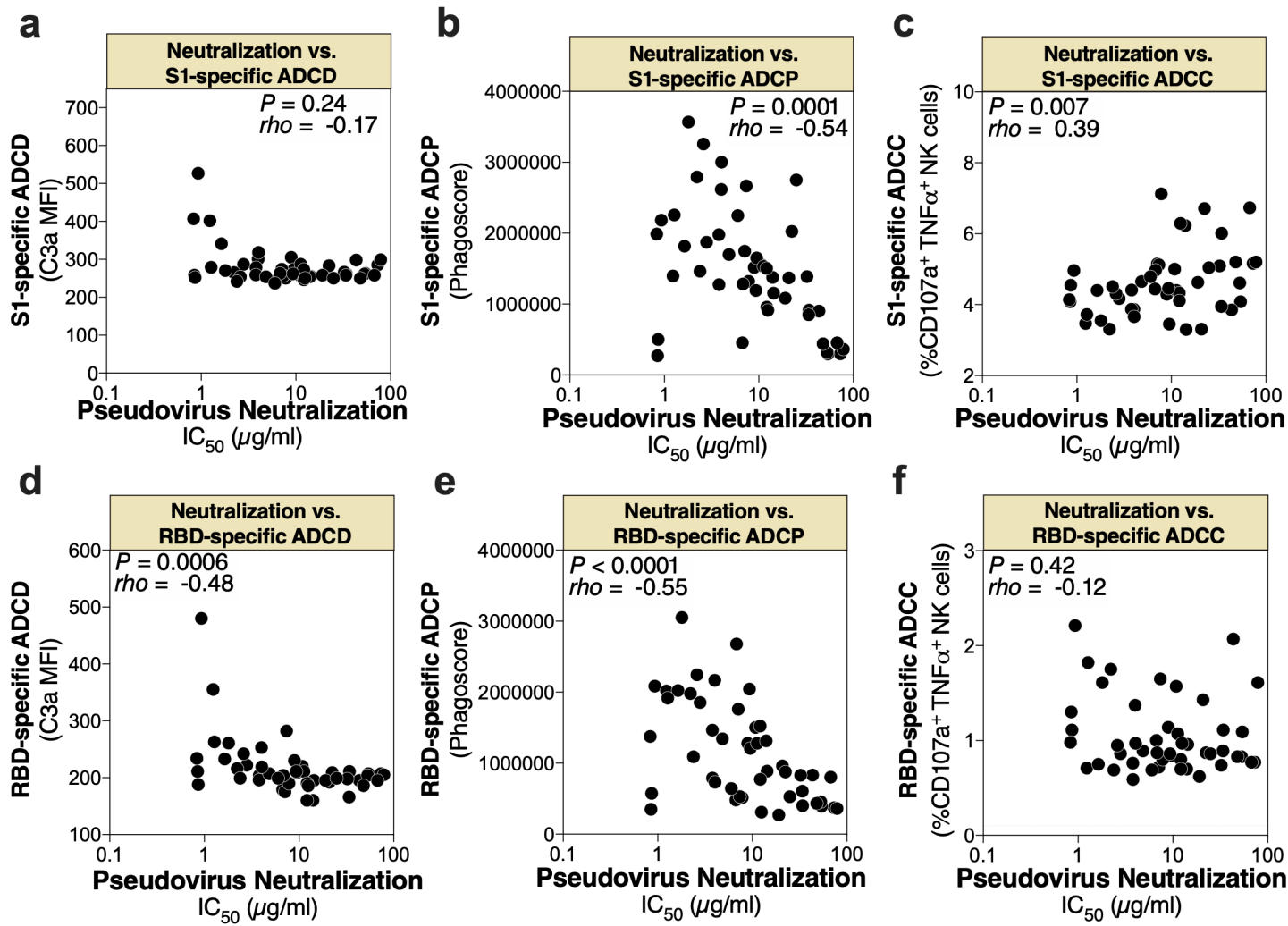

Supplement: FIG S1 [file mBio.00281-21-sf001.pdf]

Supplementary Figure 2

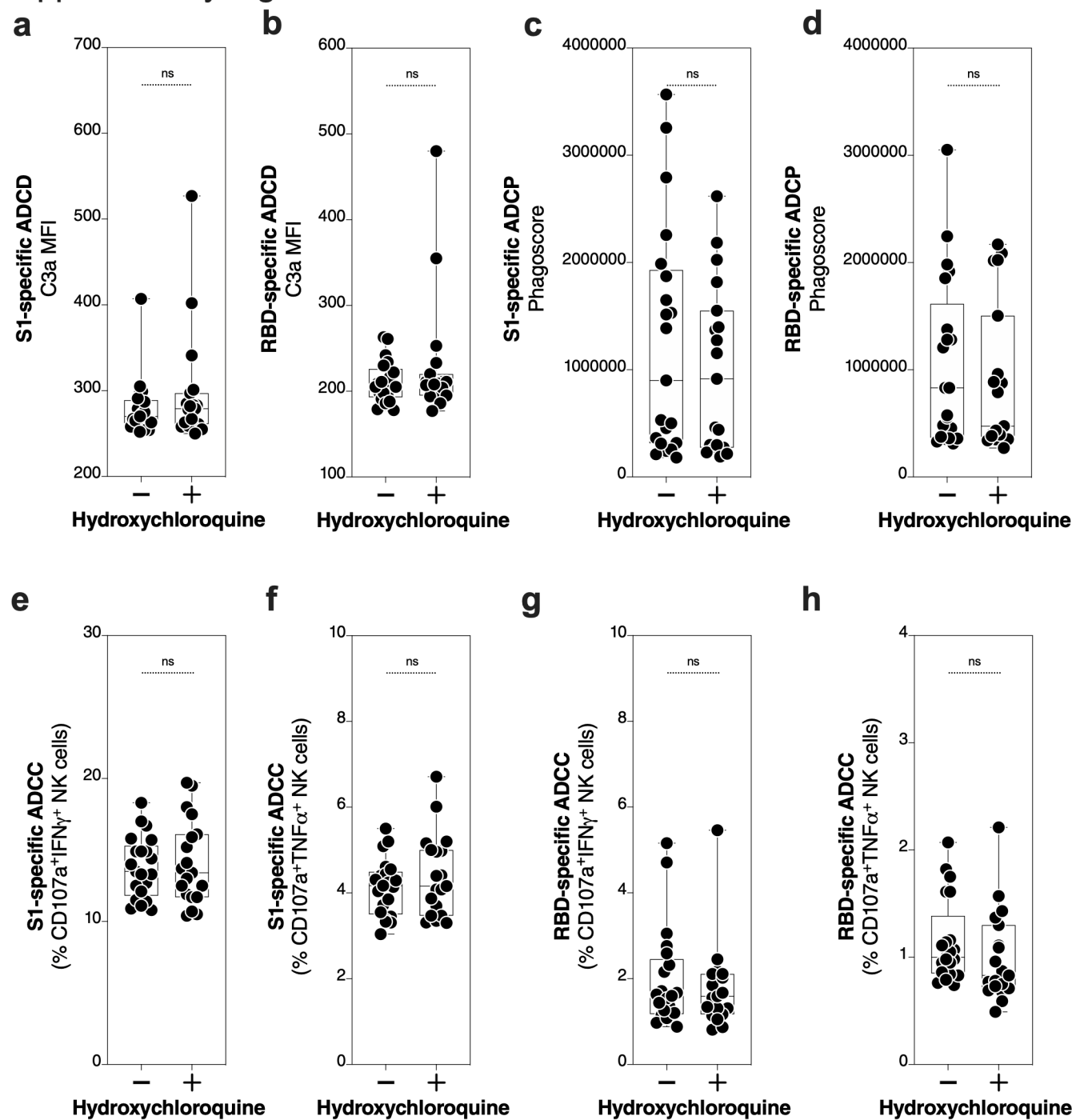

Supplement: FIG S2 [file mBio.00281-21-sf002.pdf]

Supplementary Figure 3

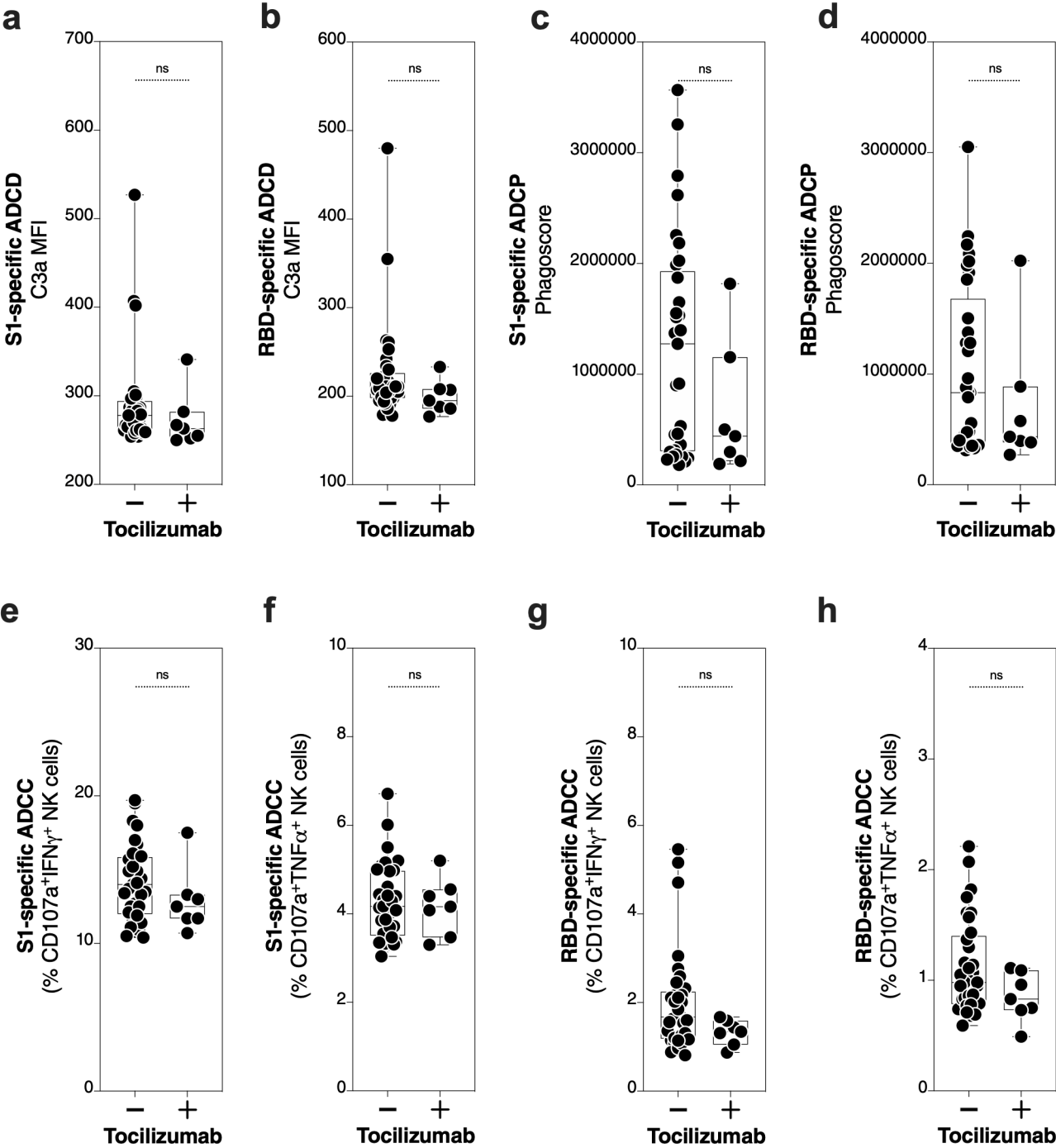

Supplement: FIG S3 [file mBio.00281-21-sf003.pdf]

Supplementary Figure 4

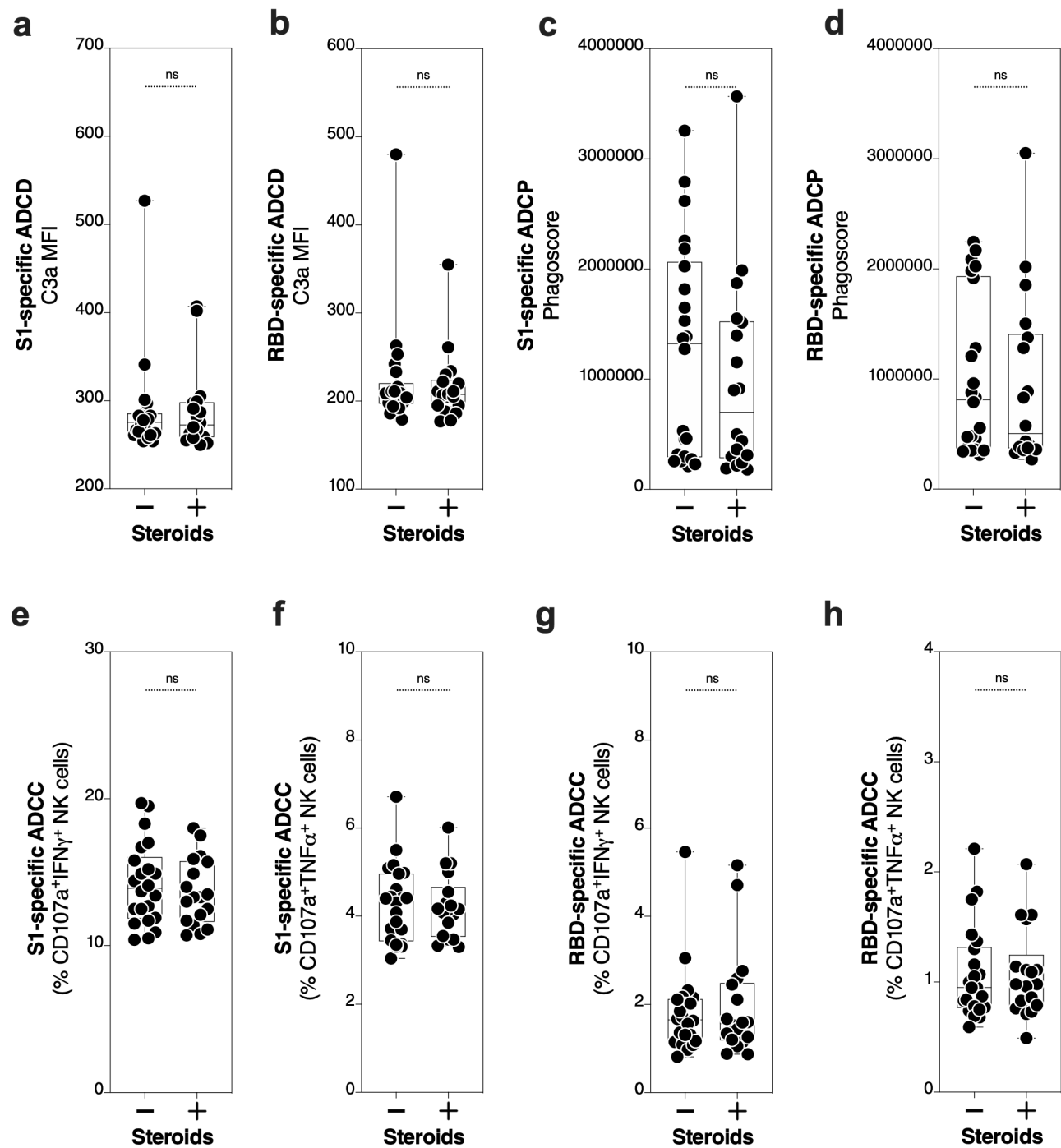

Supplement: FIG S4 [file mBio.00281-21-sf004.pdf]
