# Supplementary material for: COVID-19 Severity Is Associated with Differential Antibody Fc-Mediated Innate Immune Functions
Source: mBio. 2021 Apr 20;12(2):e00281-21. doi: 10.1128/mBio.00281-21 (PMC8092230; doi:10.1128/mBio.00281-21)
Supplement: TABLE S2 [file mBio.00281-21-st002.pdf]

Supplementary Table 2. A list of plasma markers measured in this study.

Marker	Name	Method of Measurement
CRP	C-reactive protein	ELISA; R&D Systems
IP-10	C-X-C motif chemokine ligand 10 (CXCL10)	Multiplex meso scale cytokine assay
MCP-2	Chemokine (C-C motif) ligand 8 (CCL8)	Multiplex meso scale cytokine assay
Fractalkine	chemokine (C-X3-C motif) ligand 1 (CX3CL1)	Multiplex meso scale cytokine assay
C3a	Complement component 3a	ELISA; Thermo Fischer
d-dimer	D-dimer	ELISA; Thermo Fischer
Gal-1	Galectin-1	ELISA; R&D Systems
Gal-3	Galectin-3	ELISA; R&D Systems
Gal-9	Galectin-9	ELISA; R&D Systems
GM-CSF	Granulocyte-macrophage colony-stimulating factor	Multiplex meso scale cytokine assay
GDF-15	Growth/differentiation factor 15	ELISA; R&D Systems
Zonulin	haptoglobin 2 precursor	ELISA; MyBiosorce
IFN-β	Interferon beta	Multiplex meso scale cytokine assay
IFN-γ	Interferon gamma	Multiplex meso scale cytokine assay
IFN-α2a	interferon α2a	Multiplex meso scale cytokine assay
IL-10	Interleukin 10	Multiplex meso scale cytokine assay
IL-12/IL-23p40	Interleukin 12 p70	Multiplex meso scale cytokine assay
IL-12p70	Interleukin 12 p70	Multiplex meso scale cytokine assay
IL-13	Interleukin 13	Multiplex meso scale cytokine assay
IL-1β	Interleukin 1β	Multiplex meso scale cytokine assay
IL-2	Interleukin 2	Multiplex meso scale cytokine assay
IL-21	Interleukin 21	Multiplex meso scale cytokine assay
IL-22	Interleukin 22	Multiplex meso scale cytokine assay
IL-23	Interleukin 23	Multiplex meso scale cytokine assay
IL-33	Interleukin 33	Multiplex meso scale cytokine assay
IL-4	Interleukin 4	Multiplex meso scale cytokine assay
IL-6	Interleukin 6	Multiplex meso scale cytokine assay
IL-15	Interleukin-12/interleukin 23 p40	Multiplex meso scale cytokine assay
I-FABP	Intestinal fatty-acid binding protein	ELISA; R&D Systems
LBP	Lipopolysaccharide binding protein	ELISA; R&D Systems
MIP-1α	Macrophage inflammatory protein alpha	Multiplex meso scale cytokine assay
MPO	Neutrophil myeloperoxidase	ELISA; Thermo Fischer
OCLN	Occludin	ELISA; Biomatik
Reg3A	Regenerating Family Member 3 Alpha	ELISA; RayBiotech
sCD14	Soulble CD14	ELISA; R&D Systems
sCD163	Soulble CD163	ELISA; R&D Systems
SDF-1a	stromal cell-derived factor 1 (SDF1) or C-X-C motif chemokine 12 (CXCL12)	Multiplex meso scale cytokine assay
TNF-α	tumor necrosis factor alpha	Multiplex meso scale cytokine assay
β-D-glucan	β-D-glucan	Limulus Amebocyte Lysate (LAL) assay; Glucatell Kit, CapeCod
