# Supplementary material for: COVID-19 Severity Is Associated with Differential Antibody Fc-Mediated Innate Immune Functions
Source: mBio. 2021 Apr 20;12(2):e00281-21. doi: 10.1128/mBio.00281-21 (PMC8092230; doi:10.1128/mBio.00281-21)
Supplement: TABLE S1 [file mBio.00281-21-st001.pdf]

Supplementary Table 1. Demographic and clinical characteristics of the study cohort

		SARS-CoV2 Negative -	SARS-CoV2 Positive outpatients	SARS-CoV2 Positive Inpatients	*P* value** Outpatients vs inpatients
number *(n)*		20	20	40	-
Female, *n* (%)		10 (50)	12 (60)	18 (45)	0.41
Age, years, median (IQR)		55.5 (15.6)	52.5 (12.75)	58.5 (6)	-
Body mass index (BMI)					
	Normal Weight (<25), %	-	6.7*	15	0.66
	Overweight/obese (>25), %	-	93.3*	85	0.66
Pre-diabetes, %		-	6.7*	22.5	0.25
Diabetes Mellitus (DM), %		-	6.7*	42.5	0.01
High blood pressure, %		-	33.3*	60	0.13
Asthma,%		-	13.3*	10	0.66
Hydroxychloroquine, %		-	6.7*	47.5	-
Remdesivir, %		-	-	15	-
Tocilizumab, %		-	-	17.5	-
Chronic Steroid Use, %		-	-	15	-
Acute Steroid Use, %		-	-	30	-
Plasma IV nutrition, %		-	-	0	-
Enteral nutrition use, %		-	-	40	-
Antibiotic administration, %		-	-	72.5	-
Ethnicity		-	-		
	African American, *n* (%)	0	0	16 (40)	-
	Hispanic or Latino	0	2 (10.0)	15 (37.5)	-
	Caucasian, *n* (%)	3 (15.0)	13 (65.0)	7 (17.5)	-
	Other, *n* (%)	2 (10.0)	1 (5.0)	1(2.5)	-
	Unknown, *n* (%)	15 (75.0)	4 (20.0)	1 (2.5)	-

*Based on15 out of 20 donors with available data

** Fisher's exact test
